# Supplementary material for: Pyruvate Dehydrogenase Kinase 1 inhibition mediated oxidative phosphorylation enhancement in cartilage promotes osteoarthritis progression
Source: BMC Musculoskelet Disord. 2023 Jul 20;24:597. doi: 10.1186/s12891-023-06585-6 (PMC10357736; doi:10.1186/s12891-023-06585-6)
Supplement: Supplementary file 1 — Additional file 1. [file 12891_2023_6585_MOESM1_ESM.zip › BP.Gsea.1653623667859/GOBP_RRNA_METABOLIC_PROCESS.html]

Details for gene set GOBP\_RRNA\_METABOLIC\_PROCESS[GSEA]

|  || Dataset | 55457\_collapsed\_to\_symbols.cls.cls #OA\_versus\_normal.cls.cls #OA\_versus\_normal\_repos |
| Phenotype | cls.cls#OA\_versus\_normal\_repos |
| Upregulated in class | normal |
| GeneSet | GOBP\_RRNA\_METABOLIC\_PROCESS |
| Enrichment Score (ES) | -0.3780816 |
| Normalized Enrichment Score (NES) | -1.8148392 |
| Nominal p-value | 0.0 |
| FDR q-value | 0.08881878 |
| FWER p-Value | 0.966 |
Table: GSEA Results Summary

  

Fig 1: Enrichment plot: GOBP\_RRNA\_METABOLIC\_PROCESS      
 Profile of the Running ES Score & Positions of GeneSet Members on the Rank Ordered List

  

| SYMBOL | TITLE | RANK IN GENE LIST | RANK METRIC SCORE | RUNNING ES | CORE ENRICHMENT || 1 | NVL | nuclear VCP like [Source:HGNC Symbol;Acc:HGNC:8070] | 73 | 0.956 | 0.0077 | No |
| 2 | BUD23 | BUD23 rRNA methyltransferase and ribosome maturation factor [Source:HGNC Symbol;Acc:HGNC:16405] | 210 | 0.813 | 0.0084 | No |
| 3 | RPP30 | ribonuclease P/MRP subunit p30 [Source:HGNC Symbol;Acc:HGNC:17688] | 265 | 0.777 | 0.0151 | No |
| 4 | IMP3 | IMP U3 small nucleolar ribonucleoprotein 3 [Source:HGNC Symbol;Acc:HGNC:14497] | 381 | 0.714 | 0.0161 | No |
| 5 | POP5 | "POP5 homolog, ribonuclease P/MRP subunit [Source:HGNC Symbol;Acc:HGNC:17689]" | 494 | 0.662 | 0.0165 | No |
| 6 | METTL18 | methyltransferase like 18 [Source:HGNC Symbol;Acc:HGNC:28793] | 685 | 0.597 | 0.0100 | No |
| 7 | NPM3 | nucleophosmin/nucleoplasmin 3 [Source:HGNC Symbol;Acc:HGNC:7931] | 868 | 0.551 | 0.0034 | No |
| 8 | POP4 | "POP4 homolog, ribonuclease P/MRP subunit [Source:HGNC Symbol;Acc:HGNC:30081]" | 1015 | 0.514 | -0.0009 | No |
| 9 | GTF2H5 | general transcription factor IIH subunit 5 [Source:HGNC Symbol;Acc:HGNC:21157] | 1032 | 0.511 | 0.0050 | No |
| 10 | RPF1 | ribosome production factor 1 homolog [Source:HGNC Symbol;Acc:HGNC:30350] | 1061 | 0.504 | 0.0099 | No |
| 11 | MRPS11 | mitochondrial ribosomal protein S11 [Source:HGNC Symbol;Acc:HGNC:14050] | 1305 | 0.457 | -0.0028 | No |
| 12 | POP7 | "POP7 homolog, ribonuclease P/MRP subunit [Source:HGNC Symbol;Acc:HGNC:19949]" | 1337 | 0.452 | 0.0011 | No |
| 13 | TRMT112 | tRNA methyltransferase activator subunit 11-2 [Source:HGNC Symbol;Acc:HGNC:26940] | 1359 | 0.449 | 0.0057 | No |
| 14 | NSA2 | NSA2 ribosome biogenesis factor [Source:HGNC Symbol;Acc:HGNC:30728] | 1508 | 0.428 | 0.0001 | No |
| 15 | GTF3C2 | general transcription factor IIIC subunit 2 [Source:HGNC Symbol;Acc:HGNC:4665] | 1583 | 0.416 | 0.0001 | No |
| 16 | ZNHIT3 | zinc finger HIT-type containing 3 [Source:HGNC Symbol;Acc:HGNC:12309] | 1648 | 0.407 | 0.0008 | No |
| 17 | RRP8 | ribosomal RNA processing 8 [Source:HGNC Symbol;Acc:HGNC:29030] | 1673 | 0.402 | 0.0045 | No |
| 18 | EMG1 | EMG1 N1-specific pseudouridine methyltransferase [Source:HGNC Symbol;Acc:HGNC:16912] | 1688 | 0.401 | 0.0091 | No |
| 19 | C1D | C1D nuclear receptor corepressor [Source:HGNC Symbol;Acc:HGNC:29911] | 1848 | 0.379 | 0.0019 | No |
| 20 | MRM2 | mitochondrial rRNA methyltransferase 2 [Source:HGNC Symbol;Acc:HGNC:16352] | 1880 | 0.375 | 0.0047 | No |
| 21 | EXOSC7 | exosome component 7 [Source:HGNC Symbol;Acc:HGNC:28112] | 1969 | 0.364 | 0.0029 | No |
| 22 | ANG | angiogenin [Source:HGNC Symbol;Acc:HGNC:483] | 2009 | 0.360 | 0.0049 | No |
| 23 | NHP2 | NHP2 ribonucleoprotein [Source:HGNC Symbol;Acc:HGNC:14377] | 2026 | 0.358 | 0.0087 | No |
| 24 | DROSHA | drosha ribonuclease III [Source:HGNC Symbol;Acc:HGNC:17904] | 2054 | 0.356 | 0.0115 | No |
| 25 | NOP10 | NOP10 ribonucleoprotein [Source:HGNC Symbol;Acc:HGNC:14378] | 2068 | 0.354 | 0.0155 | No |
| 26 | METTL5 | "methyltransferase 5, N6-adenosine [Source:HGNC Symbol;Acc:HGNC:25006]" | 2107 | 0.349 | 0.0174 | No |
| 27 | MTREX | Mtr4 exosome RNA helicase [Source:HGNC Symbol;Acc:HGNC:18734] | 2138 | 0.346 | 0.0199 | No |
| 28 | GTF3A | general transcription factor IIIA [Source:HGNC Symbol;Acc:HGNC:4662] | 2151 | 0.344 | 0.0238 | No |
| 29 | TBL3 | transducin beta like 3 [Source:HGNC Symbol;Acc:HGNC:11587] | 2154 | 0.344 | 0.0285 | No |
| 30 | UTP14C | UTP14C small subunit processome component [Source:HGNC Symbol;Acc:HGNC:20321] | 2336 | 0.322 | 0.0187 | No |
| 31 | PIH1D1 | PIH1 domain containing 1 [Source:HGNC Symbol;Acc:HGNC:26075] | 2667 | 0.283 | -0.0033 | No |
| 32 | TSR2 | TSR2 ribosome maturation factor [Source:HGNC Symbol;Acc:HGNC:25455] | 2924 | 0.258 | -0.0198 | No |
| 33 | RPUSD2 | RNA pseudouridine synthase domain containing 2 [Source:HGNC Symbol;Acc:HGNC:24180] | 2934 | 0.257 | -0.0169 | No |
| 34 | REXO4 | "REX4 homolog, 3'-5' exonuclease [Source:HGNC Symbol;Acc:HGNC:12820]" | 2953 | 0.255 | -0.0148 | No |
| 35 | FRG1 | FSHD region gene 1 [Source:HGNC Symbol;Acc:HGNC:3954] | 3254 | 0.226 | -0.0352 | No |
| 36 | CAVIN1 | caveolae associated protein 1 [Source:HGNC Symbol;Acc:HGNC:9688] | 3271 | 0.224 | -0.0333 | No |
| 37 | MRPL44 | mitochondrial ribosomal protein L44 [Source:HGNC Symbol;Acc:HGNC:16650] | 3306 | 0.220 | -0.0329 | No |
| 38 | TAF1B | "TATA-box binding protein associated factor, RNA polymerase I subunit B [Source:HGNC Symbol;Acc:HGNC:11533]" | 3496 | 0.203 | -0.0450 | No |
| 39 | ERCC2 | "ERCC excision repair 2, TFIIH core complex helicase subunit [Source:HGNC Symbol;Acc:HGNC:3434]" | 3544 | 0.198 | -0.0459 | No |
| 40 | NSUN3 | NOP2/Sun RNA methyltransferase 3 [Source:HGNC Symbol;Acc:HGNC:26208] | 3612 | 0.192 | -0.0485 | No |
| 41 | RIOK2 | RIO kinase 2 [Source:HGNC Symbol;Acc:HGNC:18999] | 3615 | 0.192 | -0.0459 | No |
| 42 | MACROH2A2 | macroH2A.2 histone [Source:HGNC Symbol;Acc:HGNC:14453] | 3793 | 0.178 | -0.0574 | No |
| 43 | SNU13 | small nuclear ribonucleoprotein 13 [Source:HGNC Symbol;Acc:HGNC:7819] | 3795 | 0.178 | -0.0549 | No |
| 44 | MAPT | microtubule associated protein tau [Source:HGNC Symbol;Acc:HGNC:6893] | 3976 | 0.158 | -0.0669 | No |
| 45 | LSM6 | "LSM6 homolog, U6 small nuclear RNA and mRNA degradation associated [Source:HGNC Symbol;Acc:HGNC:17017]" | 4019 | 0.154 | -0.0681 | No |
| 46 | DIS3 | "DIS3 homolog, exosome endoribonuclease and 3'-5' exoribonuclease [Source:HGNC Symbol;Acc:HGNC:20604]" | 4187 | 0.139 | -0.0793 | No |
| 47 | RIOK3 | RIO kinase 3 [Source:HGNC Symbol;Acc:HGNC:11451] | 4206 | 0.137 | -0.0787 | No |
| 48 | FBL | fibrillarin [Source:HGNC Symbol;Acc:HGNC:3599] | 4219 | 0.135 | -0.0778 | No |
| 49 | EXOSC5 | exosome component 5 [Source:HGNC Symbol;Acc:HGNC:24662] | 4295 | 0.128 | -0.0819 | No |
| 50 | ERI3 | ERI1 exoribonuclease family member 3 [Source:HGNC Symbol;Acc:HGNC:17276] | 4627 | 0.102 | -0.1065 | No |
| 51 | ABT1 | activator of basal transcription 1 [Source:HGNC Symbol;Acc:HGNC:17369] | 4783 | 0.088 | -0.1175 | No |
| 52 | DDX47 | DEAD-box helicase 47 [Source:HGNC Symbol;Acc:HGNC:18682] | 4789 | 0.088 | -0.1167 | No |
| 53 | MRM3 | mitochondrial rRNA methyltransferase 3 [Source:HGNC Symbol;Acc:HGNC:18485] | 4806 | 0.087 | -0.1167 | No |
| 54 | KAT2B | lysine acetyltransferase 2B [Source:HGNC Symbol;Acc:HGNC:8638] | 4867 | 0.082 | -0.1203 | No |
| 55 | ZCCHC4 | zinc finger CCHC-type containing 4 [Source:HGNC Symbol;Acc:HGNC:22917] | 4909 | 0.079 | -0.1224 | No |
| 56 | UTP3 | UTP3 small subunit processome component [Source:HGNC Symbol;Acc:HGNC:24477] | 4934 | 0.077 | -0.1232 | No |
| 57 | RPL10A | ribosomal protein L10a [Source:HGNC Symbol;Acc:HGNC:10299] | 4974 | 0.074 | -0.1252 | No |
| 58 | MPHOSPH6 | M-phase phosphoprotein 6 [Source:HGNC Symbol;Acc:HGNC:7214] | 5077 | 0.067 | -0.1323 | No |
| 59 | GTF3C3 | general transcription factor IIIC subunit 3 [Source:HGNC Symbol;Acc:HGNC:4666] | 5420 | 0.039 | -0.1587 | No |
| 60 | NSUN5 | NOP2/Sun RNA methyltransferase 5 [Source:HGNC Symbol;Acc:HGNC:16385] | 5434 | 0.038 | -0.1592 | No |
| 61 | TP53 | tumor protein p53 [Source:HGNC Symbol;Acc:HGNC:11998] | 5511 | 0.032 | -0.1648 | No |
| 62 | UTP18 | UTP18 small subunit processome component [Source:HGNC Symbol;Acc:HGNC:24274] | 5522 | 0.031 | -0.1651 | No |
| 63 | PIN4 | "peptidylprolyl cis/trans isomerase, NIMA-interacting 4 [Source:HGNC Symbol;Acc:HGNC:8992]" | 5567 | 0.027 | -0.1682 | No |
| 64 | SART1 | "spliceosome associated factor 1, recruiter of U4/U6.U5 tri-snRNP [Source:HGNC Symbol;Acc:HGNC:10538]" | 5690 | 0.017 | -0.1776 | No |
| 65 | EXOSC4 | exosome component 4 [Source:HGNC Symbol;Acc:HGNC:18189] | 5693 | 0.016 | -0.1775 | No |
| 66 | TFB1M | "transcription factor B1, mitochondrial [Source:HGNC Symbol;Acc:HGNC:17037]" | 5789 | 0.009 | -0.1849 | No |
| 67 | RPS19 | ribosomal protein S19 [Source:HGNC Symbol;Acc:HGNC:10402] | 5952 | -0.002 | -0.1976 | No |
| 68 | MACROH2A1 | macroH2A.1 histone [Source:HGNC Symbol;Acc:HGNC:4740] | 5953 | -0.002 | -0.1976 | No |
| 69 | NOL11 | nucleolar protein 11 [Source:HGNC Symbol;Acc:HGNC:24557] | 5984 | -0.003 | -0.1999 | No |
| 70 | SUV39H1 | suppressor of variegation 3-9 homolog 1 [Source:HGNC Symbol;Acc:HGNC:11479] | 6028 | -0.007 | -0.2032 | No |
| 71 | MPHOSPH10 | M-phase phosphoprotein 10 [Source:HGNC Symbol;Acc:HGNC:7213] | 6088 | -0.011 | -0.2077 | No |
| 72 | EIF6 | eukaryotic translation initiation factor 6 [Source:HGNC Symbol;Acc:HGNC:6159] | 6140 | -0.015 | -0.2115 | No |
| 73 | ISG20 | interferon stimulated exonuclease gene 20 [Source:HGNC Symbol;Acc:HGNC:6130] | 6212 | -0.022 | -0.2168 | No |
| 74 | TRMT61B | tRNA methyltransferase 61B [Source:HGNC Symbol;Acc:HGNC:26070] | 6223 | -0.022 | -0.2173 | No |
| 75 | NOP53 | NOP53 ribosome biogenesis factor [Source:HGNC Symbol;Acc:HGNC:4333] | 6334 | -0.031 | -0.2255 | No |
| 76 | NGDN | neuroguidin [Source:HGNC Symbol;Acc:HGNC:20271] | 6370 | -0.033 | -0.2278 | No |
| 77 | RPL35A | ribosomal protein L35a [Source:HGNC Symbol;Acc:HGNC:10345] | 6383 | -0.034 | -0.2283 | No |
| 78 | MARS1 | methionyl-tRNA synthetase 1 [Source:HGNC Symbol;Acc:HGNC:6898] | 6400 | -0.035 | -0.2290 | No |
| 79 | RBFA | ribosome binding factor A [Source:HGNC Symbol;Acc:HGNC:26120] | 6473 | -0.040 | -0.2342 | No |
| 80 | GTF3C5 | general transcription factor IIIC subunit 5 [Source:HGNC Symbol;Acc:HGNC:4668] | 6506 | -0.043 | -0.2361 | No |
| 81 | NOL8 | nucleolar protein 8 [Source:HGNC Symbol;Acc:HGNC:23387] | 6709 | -0.056 | -0.2512 | No |
| 82 | METTL25B | methyltransferase like 25B [Source:HGNC Symbol;Acc:HGNC:24273] | 6774 | -0.061 | -0.2554 | No |
| 83 | RPP38 | ribonuclease P/MRP subunit p38 [Source:HGNC Symbol;Acc:HGNC:30329] | 6814 | -0.064 | -0.2576 | No |
| 84 | MTERF4 | mitochondrial transcription termination factor 4 [Source:HGNC Symbol;Acc:HGNC:28785] | 6972 | -0.078 | -0.2688 | No |
| 85 | FCF1 | FCF1 rRNA-processing protein [Source:HGNC Symbol;Acc:HGNC:20220] | 6999 | -0.080 | -0.2698 | No |
| 86 | UTP11 | UTP11 small subunit processome component [Source:HGNC Symbol;Acc:HGNC:24329] | 7015 | -0.081 | -0.2698 | No |
| 87 | TFB2M | "transcription factor B2, mitochondrial [Source:HGNC Symbol;Acc:HGNC:18559]" | 7112 | -0.089 | -0.2761 | No |
| 88 | GTF3C1 | general transcription factor IIIC subunit 1 [Source:HGNC Symbol;Acc:HGNC:4664] | 7124 | -0.090 | -0.2757 | No |
| 89 | WBP11 | WW domain binding protein 11 [Source:HGNC Symbol;Acc:HGNC:16461] | 7312 | -0.103 | -0.2890 | No |
| 90 | DCAF13 | DDB1 and CUL4 associated factor 13 [Source:HGNC Symbol;Acc:HGNC:24535] | 7459 | -0.115 | -0.2989 | No |
| 91 | RPL35 | ribosomal protein L35 [Source:HGNC Symbol;Acc:HGNC:10344] | 7563 | -0.124 | -0.3053 | No |
| 92 | ERN2 | endoplasmic reticulum to nucleus signaling 2 [Source:HGNC Symbol;Acc:HGNC:16942] | 7565 | -0.124 | -0.3036 | No |
| 93 | BRF1 | BRF1 RNA polymerase III transcription initiation factor subunit [Source:HGNC Symbol;Acc:HGNC:11551] | 7598 | -0.127 | -0.3043 | No |
| 94 | IMP4 | IMP U3 small nucleolar ribonucleoprotein 4 [Source:HGNC Symbol;Acc:HGNC:30856] | 7643 | -0.130 | -0.3060 | No |
| 95 | EIF4A3 | eukaryotic translation initiation factor 4A3 [Source:HGNC Symbol;Acc:HGNC:18683] | 7646 | -0.130 | -0.3043 | No |
| 96 | TSR3 | TSR3 ribosome maturation factor [Source:HGNC Symbol;Acc:HGNC:14175] | 7676 | -0.133 | -0.3047 | No |
| 97 | NOL10 | nucleolar protein 10 [Source:HGNC Symbol;Acc:HGNC:25862] | 7880 | -0.146 | -0.3187 | No |
| 98 | DDX49 | DEAD-box helicase 49 [Source:HGNC Symbol;Acc:HGNC:18684] | 7945 | -0.151 | -0.3216 | No |
| 99 | DDX18 | DEAD-box helicase 18 [Source:HGNC Symbol;Acc:HGNC:2741] | 7996 | -0.154 | -0.3234 | No |
| 100 | NOL6 | nucleolar protein 6 [Source:HGNC Symbol;Acc:HGNC:19910] | 8081 | -0.161 | -0.3277 | No |
| 101 | MAK16 | MAK16 homolog [Source:HGNC Symbol;Acc:HGNC:13703] | 8098 | -0.163 | -0.3267 | No |
| 102 | KRR1 | KRR1 small subunit processome component homolog [Source:HGNC Symbol;Acc:HGNC:5176] | 8126 | -0.165 | -0.3265 | No |
| 103 | NOC4L | nucleolar complex associated 4 homolog [Source:HGNC Symbol;Acc:HGNC:28461] | 8225 | -0.173 | -0.3318 | No |
| 104 | RSL1D1 | ribosomal L1 domain containing 1 [Source:HGNC Symbol;Acc:HGNC:24534] | 8286 | -0.177 | -0.3340 | No |
| 105 | RPP40 | ribonuclease P/MRP subunit p40 [Source:HGNC Symbol;Acc:HGNC:20992] | 8318 | -0.179 | -0.3339 | No |
| 106 | RPS21 | ribosomal protein S21 [Source:HGNC Symbol;Acc:HGNC:10409] | 8394 | -0.185 | -0.3372 | No |
| 107 | EXOSC10 | exosome component 10 [Source:HGNC Symbol;Acc:HGNC:9138] | 8404 | -0.186 | -0.3353 | No |
| 108 | WDR12 | WD repeat domain 12 [Source:HGNC Symbol;Acc:HGNC:14098] | 8505 | -0.194 | -0.3405 | No |
| 109 | EXOSC8 | exosome component 8 [Source:HGNC Symbol;Acc:HGNC:17035] | 8554 | -0.198 | -0.3415 | No |
| 110 | UTP6 | UTP6 small subunit processome component [Source:HGNC Symbol;Acc:HGNC:18279] | 8638 | -0.203 | -0.3452 | No |
| 111 | UTP25 | UTP25 small subunit processor component [Source:HGNC Symbol;Acc:HGNC:28440] | 8699 | -0.209 | -0.3470 | No |
| 112 | PWP1 | "PWP1 homolog, endonuclein [Source:HGNC Symbol;Acc:HGNC:17015]" | 8724 | -0.211 | -0.3459 | No |
| 113 | SIRT7 | sirtuin 7 [Source:HGNC Symbol;Acc:HGNC:14935] | 8733 | -0.211 | -0.3435 | No |
| 114 | DEDD | death effector domain containing [Source:HGNC Symbol;Acc:HGNC:2755] | 8888 | -0.224 | -0.3525 | No |
| 115 | RRP1B | ribosomal RNA processing 1B [Source:HGNC Symbol;Acc:HGNC:23818] | 8909 | -0.225 | -0.3509 | No |
| 116 | EBNA1BP2 | EBNA1 binding protein 2 [Source:HGNC Symbol;Acc:HGNC:15531] | 8931 | -0.226 | -0.3494 | No |
| 117 | NSUN5P1 | NSUN5 pseudogene 1 [Source:HGNC Symbol;Acc:HGNC:19146] | 8988 | -0.231 | -0.3506 | No |
| 118 | MRM1 | mitochondrial rRNA methyltransferase 1 [Source:HGNC Symbol;Acc:HGNC:26202] | 9114 | -0.241 | -0.3571 | No |
| 119 | FTSJ3 | FtsJ RNA 2'-O-methyltransferase 3 [Source:HGNC Symbol;Acc:HGNC:17136] | 9193 | -0.247 | -0.3597 | No |
| 120 | BYSL | bystin like [Source:HGNC Symbol;Acc:HGNC:1157] | 9277 | -0.254 | -0.3627 | No |
| 121 | RPS25 | ribosomal protein S25 [Source:HGNC Symbol;Acc:HGNC:10413] | 9282 | -0.254 | -0.3594 | No |
| 122 | HEATR1 | HEAT repeat containing 1 [Source:HGNC Symbol;Acc:HGNC:25517] | 9339 | -0.259 | -0.3602 | No |
| 123 | UTP14A | UTP14A small subunit processome component [Source:HGNC Symbol;Acc:HGNC:10665] | 9536 | -0.277 | -0.3718 | No |
| 124 | IPPK | inositol-pentakisphosphate 2-kinase [Source:HGNC Symbol;Acc:HGNC:14645] | 9547 | -0.278 | -0.3687 | No |
| 125 | RCL1 | RNA terminal phosphate cyclase like 1 [Source:HGNC Symbol;Acc:HGNC:17687] | 9586 | -0.282 | -0.3677 | No |
| 126 | PRKDC | "protein kinase, DNA-activated, catalytic subunit [Source:HGNC Symbol;Acc:HGNC:9413]" | 9632 | -0.286 | -0.3672 | No |
| 127 | TCOF1 | treacle ribosome biogenesis factor 1 [Source:HGNC Symbol;Acc:HGNC:11654] | 9771 | -0.298 | -0.3739 | Yes |
| 128 | RRP15 | ribosomal RNA processing 15 homolog [Source:HGNC Symbol;Acc:HGNC:24255] | 9775 | -0.299 | -0.3699 | Yes |
| 129 | WDR74 | WD repeat domain 74 [Source:HGNC Symbol;Acc:HGNC:25529] | 9777 | -0.299 | -0.3658 | Yes |
| 130 | PELO | pelota mRNA surveillance and ribosome rescue factor [Source:HGNC Symbol;Acc:HGNC:8829] | 9806 | -0.302 | -0.3638 | Yes |
| 131 | URB1 | URB1 ribosome biogenesis homolog [Source:HGNC Symbol;Acc:HGNC:17344] | 9833 | -0.304 | -0.3615 | Yes |
| 132 | RNASEL | ribonuclease L [Source:HGNC Symbol;Acc:HGNC:10050] | 9965 | -0.317 | -0.3674 | Yes |
| 133 | SMARCA4 | "SWI/SNF related, matrix associated, actin dependent regulator of chromatin, subfamily a, member 4 [Source:HGNC Symbol;Acc:HGNC:11100]" | 10009 | -0.322 | -0.3662 | Yes |
| 134 | DIMT1 | DIM1 rRNA methyltransferase and ribosome maturation factor [Source:HGNC Symbol;Acc:HGNC:30217] | 10023 | -0.324 | -0.3627 | Yes |
| 135 | SHQ1 | "SHQ1, H/ACA ribonucleoprotein assembly factor [Source:HGNC Symbol;Acc:HGNC:25543]" | 10045 | -0.326 | -0.3598 | Yes |
| 136 | RRP9 | "ribosomal RNA processing 9, U3 small nucleolar RNA binding protein [Source:HGNC Symbol;Acc:HGNC:16829]" | 10064 | -0.327 | -0.3566 | Yes |
| 137 | GEMIN4 | gem nuclear organelle associated protein 4 [Source:HGNC Symbol;Acc:HGNC:15717] | 10075 | -0.328 | -0.3528 | Yes |
| 138 | SMARCB1 | "SWI/SNF related, matrix associated, actin dependent regulator of chromatin, subfamily b, member 1 [Source:HGNC Symbol;Acc:HGNC:11103]" | 10117 | -0.333 | -0.3513 | Yes |
| 139 | DDX51 | DEAD-box helicase 51 [Source:HGNC Symbol;Acc:HGNC:20082] | 10133 | -0.334 | -0.3478 | Yes |
| 140 | RPL5 | ribosomal protein L5 [Source:HGNC Symbol;Acc:HGNC:10360] | 10201 | -0.341 | -0.3483 | Yes |
| 141 | ZNHIT6 | zinc finger HIT-type containing 6 [Source:HGNC Symbol;Acc:HGNC:26089] | 10220 | -0.343 | -0.3449 | Yes |
| 142 | PES1 | pescadillo ribosomal biogenesis factor 1 [Source:HGNC Symbol;Acc:HGNC:8848] | 10268 | -0.347 | -0.3437 | Yes |
| 143 | BRIX1 | biogenesis of ribosomes BRX1 [Source:HGNC Symbol;Acc:HGNC:24170] | 10275 | -0.348 | -0.3393 | Yes |
| 144 | MRTO4 | "MRT4 homolog, ribosome maturation factor [Source:HGNC Symbol;Acc:HGNC:18477]" | 10300 | -0.350 | -0.3363 | Yes |
| 145 | NOL9 | nucleolar protein 9 [Source:HGNC Symbol;Acc:HGNC:26265] | 10361 | -0.357 | -0.3360 | Yes |
| 146 | RRP7A | ribosomal RNA processing 7 homolog A [Source:HGNC Symbol;Acc:HGNC:24286] | 10449 | -0.367 | -0.3377 | Yes |
| 147 | RPS8 | ribosomal protein S8 [Source:HGNC Symbol;Acc:HGNC:10441] | 10525 | -0.376 | -0.3383 | Yes |
| 148 | RPS14 | ribosomal protein S14 [Source:HGNC Symbol;Acc:HGNC:10387] | 10558 | -0.379 | -0.3355 | Yes |
| 149 | DKC1 | dyskerin pseudouridine synthase 1 [Source:HGNC Symbol;Acc:HGNC:2890] | 10591 | -0.382 | -0.3326 | Yes |
| 150 | PA2G4 | proliferation-associated 2G4 [Source:HGNC Symbol;Acc:HGNC:8550] | 10598 | -0.383 | -0.3277 | Yes |
| 151 | POLR1G | RNA polymerase I subunit G [Source:HGNC Symbol;Acc:HGNC:24219] | 10599 | -0.383 | -0.3223 | Yes |
| 152 | EXOSC9 | exosome component 9 [Source:HGNC Symbol;Acc:HGNC:9137] | 10606 | -0.384 | -0.3174 | Yes |
| 153 | RPS7 | ribosomal protein S7 [Source:HGNC Symbol;Acc:HGNC:10440] | 10608 | -0.384 | -0.3121 | Yes |
| 154 | RPP25 | ribonuclease P and MRP subunit p25 [Source:HGNC Symbol;Acc:HGNC:30361] | 10621 | -0.385 | -0.3076 | Yes |
| 155 | WDR18 | WD repeat domain 18 [Source:HGNC Symbol;Acc:HGNC:17956] | 10685 | -0.391 | -0.3071 | Yes |
| 156 | NOP14 | NOP14 nucleolar protein [Source:HGNC Symbol;Acc:HGNC:16821] | 10747 | -0.398 | -0.3063 | Yes |
| 157 | RPL7A | ribosomal protein L7a [Source:HGNC Symbol;Acc:HGNC:10364] | 10811 | -0.406 | -0.3055 | Yes |
| 158 | NOLC1 | nucleolar and coiled-body phosphoprotein 1 [Source:HGNC Symbol;Acc:HGNC:15608] | 10942 | -0.423 | -0.3098 | Yes |
| 159 | RRP12 | ribosomal RNA processing 12 homolog [Source:HGNC Symbol;Acc:HGNC:29100] | 10970 | -0.427 | -0.3059 | Yes |
| 160 | NAT10 | N-acetyltransferase 10 [Source:HGNC Symbol;Acc:HGNC:29830] | 10998 | -0.429 | -0.3020 | Yes |
| 161 | DDX10 | DEAD-box helicase 10 [Source:HGNC Symbol;Acc:HGNC:2735] | 11029 | -0.434 | -0.2983 | Yes |
| 162 | RRS1 | ribosome biogenesis regulator 1 homolog [Source:HGNC Symbol;Acc:HGNC:17083] | 11045 | -0.437 | -0.2933 | Yes |
| 163 | DDX21 | DExD-box helicase 21 [Source:HGNC Symbol;Acc:HGNC:2744] | 11120 | -0.446 | -0.2929 | Yes |
| 164 | RPL14 | ribosomal protein L14 [Source:HGNC Symbol;Acc:HGNC:10305] | 11154 | -0.451 | -0.2891 | Yes |
| 165 | RPL11 | ribosomal protein L11 [Source:HGNC Symbol;Acc:HGNC:10301] | 11252 | -0.464 | -0.2903 | Yes |
| 166 | POLR1E | RNA polymerase I subunit E [Source:HGNC Symbol;Acc:HGNC:17631] | 11274 | -0.468 | -0.2853 | Yes |
| 167 | ERI2 | ERI1 exoribonuclease family member 2 [Source:HGNC Symbol;Acc:HGNC:30541] | 11320 | -0.474 | -0.2822 | Yes |
| 168 | METTL16 | "methyltransferase 16, N6-methyladenosine [Source:HGNC Symbol;Acc:HGNC:28484]" | 11346 | -0.478 | -0.2775 | Yes |
| 169 | DDX56 | DEAD-box helicase 56 [Source:HGNC Symbol;Acc:HGNC:18193] | 11386 | -0.484 | -0.2737 | Yes |
| 170 | WDR3 | WD repeat domain 3 [Source:HGNC Symbol;Acc:HGNC:12755] | 11429 | -0.492 | -0.2701 | Yes |
| 171 | LAS1L | LAS1 like ribosome biogenesis factor [Source:HGNC Symbol;Acc:HGNC:25726] | 11474 | -0.498 | -0.2666 | Yes |
| 172 | DDX52 | DExD-box helicase 52 [Source:HGNC Symbol;Acc:HGNC:20038] | 11484 | -0.499 | -0.2603 | Yes |
| 173 | USP36 | ubiquitin specific peptidase 36 [Source:HGNC Symbol;Acc:HGNC:20062] | 11560 | -0.508 | -0.2590 | Yes |
| 174 | PWP2 | PWP2 small subunit processome component [Source:HGNC Symbol;Acc:HGNC:9711] | 11604 | -0.516 | -0.2552 | Yes |
| 175 | WDR55 | WD repeat domain 55 [Source:HGNC Symbol;Acc:HGNC:25971] | 11605 | -0.517 | -0.2479 | Yes |
| 176 | GTF3C4 | general transcription factor IIIC subunit 4 [Source:HGNC Symbol;Acc:HGNC:4667] | 11621 | -0.520 | -0.2418 | Yes |
| 177 | UTP20 | UTP20 small subunit processome component [Source:HGNC Symbol;Acc:HGNC:17897] | 11656 | -0.526 | -0.2370 | Yes |
| 178 | RPS6 | ribosomal protein S6 [Source:HGNC Symbol;Acc:HGNC:10429] | 11662 | -0.526 | -0.2300 | Yes |
| 179 | RPS15 | ribosomal protein S15 [Source:HGNC Symbol;Acc:HGNC:10388] | 11671 | -0.527 | -0.2233 | Yes |
| 180 | WDR46 | WD repeat domain 46 [Source:HGNC Symbol;Acc:HGNC:13923] | 11683 | -0.529 | -0.2167 | Yes |
| 181 | DDX17 | DEAD-box helicase 17 [Source:HGNC Symbol;Acc:HGNC:2740] | 11716 | -0.535 | -0.2117 | Yes |
| 182 | RPS16 | ribosomal protein S16 [Source:HGNC Symbol;Acc:HGNC:10396] | 11755 | -0.541 | -0.2071 | Yes |
| 183 | RPL27 | ribosomal protein L27 [Source:HGNC Symbol;Acc:HGNC:10328] | 11765 | -0.544 | -0.2001 | Yes |
| 184 | PDCD11 | programmed cell death 11 [Source:HGNC Symbol;Acc:HGNC:13408] | 11847 | -0.559 | -0.1986 | Yes |
| 185 | GTPBP4 | GTP binding protein 4 [Source:HGNC Symbol;Acc:HGNC:21535] | 11861 | -0.562 | -0.1918 | Yes |
| 186 | GAR1 | GAR1 ribonucleoprotein [Source:HGNC Symbol;Acc:HGNC:14264] | 11883 | -0.568 | -0.1854 | Yes |
| 187 | NOP56 | NOP56 ribonucleoprotein [Source:HGNC Symbol;Acc:HGNC:15911] | 11939 | -0.580 | -0.1816 | Yes |
| 188 | DDX54 | DEAD-box helicase 54 [Source:HGNC Symbol;Acc:HGNC:20084] | 11969 | -0.587 | -0.1756 | Yes |
| 189 | MDN1 | midasin AAA ATPase 1 [Source:HGNC Symbol;Acc:HGNC:18302] | 11973 | -0.588 | -0.1676 | Yes |
| 190 | RRP1 | ribosomal RNA processing 1 [Source:HGNC Symbol;Acc:HGNC:18785] | 11996 | -0.592 | -0.1610 | Yes |
| 191 | PPAN | peter pan homolog [Source:HGNC Symbol;Acc:HGNC:9227] | 12022 | -0.597 | -0.1546 | Yes |
| 192 | MTOR | mechanistic target of rapamycin kinase [Source:HGNC Symbol;Acc:HGNC:3942] | 12047 | -0.605 | -0.1479 | Yes |
| 193 | RPS27 | ribosomal protein S27 [Source:HGNC Symbol;Acc:HGNC:10416] | 12071 | -0.612 | -0.1411 | Yes |
| 194 | KRI1 | KRI1 homolog [Source:HGNC Symbol;Acc:HGNC:25769] | 12074 | -0.613 | -0.1327 | Yes |
| 195 | EXOSC2 | exosome component 2 [Source:HGNC Symbol;Acc:HGNC:17097] | 12100 | -0.620 | -0.1259 | Yes |
| 196 | RPS17 | ribosomal protein S17 [Source:HGNC Symbol;Acc:HGNC:10397] | 12130 | -0.626 | -0.1194 | Yes |
| 197 | RPS24 | ribosomal protein S24 [Source:HGNC Symbol;Acc:HGNC:10411] | 12223 | -0.652 | -0.1175 | Yes |
| 198 | NCL | nucleolin [Source:HGNC Symbol;Acc:HGNC:7667] | 12255 | -0.662 | -0.1106 | Yes |
| 199 | DDX27 | DEAD-box helicase 27 [Source:HGNC Symbol;Acc:HGNC:15837] | 12359 | -0.695 | -0.1090 | Yes |
| 200 | NOP2 | NOP2 nucleolar protein [Source:HGNC Symbol;Acc:HGNC:7867] | 12372 | -0.699 | -0.1001 | Yes |
| 201 | BMS1 | BMS1 ribosome biogenesis factor [Source:HGNC Symbol;Acc:HGNC:23505] | 12403 | -0.707 | -0.0925 | Yes |
| 202 | WDR43 | WD repeat domain 43 [Source:HGNC Symbol;Acc:HGNC:28945] | 12424 | -0.716 | -0.0840 | Yes |
| 203 | RPL7 | ribosomal protein L7 [Source:HGNC Symbol;Acc:HGNC:10363] | 12519 | -0.757 | -0.0808 | Yes |
| 204 | RPS28 | ribosomal protein S28 [Source:HGNC Symbol;Acc:HGNC:10418] | 12526 | -0.761 | -0.0705 | Yes |
| 205 | SPIN1 | spindlin 1 [Source:HGNC Symbol;Acc:HGNC:11243] | 12559 | -0.777 | -0.0621 | Yes |
| 206 | BOP1 | BOP1 ribosomal biogenesis factor [Source:HGNC Symbol;Acc:HGNC:15519] | 12580 | -0.785 | -0.0527 | Yes |
| 207 | CHD7 | chromodomain helicase DNA binding protein 7 [Source:HGNC Symbol;Acc:HGNC:20626] | 12591 | -0.791 | -0.0423 | Yes |
| 208 | DDX11 | DEAD/H-box helicase 11 [Source:HGNC Symbol;Acc:HGNC:2736] | 12607 | -0.803 | -0.0322 | Yes |
| 209 | YTHDF2 | YTH N6-methyladenosine RNA binding protein 2 [Source:HGNC Symbol;Acc:HGNC:31675] | 12694 | -0.860 | -0.0269 | Yes |
| 210 | TSR1 | TSR1 ribosome maturation factor [Source:HGNC Symbol;Acc:HGNC:25542] | 12814 | -0.999 | -0.0222 | Yes |
| 211 | ESF1 | ESF1 nucleolar pre-rRNA processing protein homolog [Source:HGNC Symbol;Acc:HGNC:15898] | 12821 | -1.008 | -0.0085 | Yes |
| 212 | POLR1B | RNA polymerase I subunit B [Source:HGNC Symbol;Acc:HGNC:20454] | 12841 | -1.045 | 0.0046 | Yes |
Table: GSEA details [plain text format]

  

Fig 2: GOBP\_RRNA\_METABOLIC\_PROCESS      
 Blue-Pink O' Gram in the Space of the Analyzed GeneSet

  

Fig 3: GOBP\_RRNA\_METABOLIC\_PROCESS: Random ES distribution      
 Gene set null distribution of ES for **GOBP\_RRNA\_METABOLIC\_PROCESS**
